# Natural Cyclophilin A Inhibitors Suppress the Growth of Cancer Stem Cells in Non-Small Cell Lung Cancer by Disrupting Crosstalk between CypA/CD147 and EGFR

**DOI:** 10.3390/ijms24119437

**Published:** 2023-05-29

**Authors:** Jang Mi Han, Sung Min Kim, Hong Lae Kim, Hee Jeong Cho, Hye Jin Jung

**Affiliations:** 1 Department of Life Science and Biochemical Engineering, Graduate School, Sun Moon University, Asan 31460, Republic of Korea; gkswkdal200@naver.com (J.M.H.); tjdals8855@gmail.com (S.M.K.); tjdrjdh@naver.com (H.J.C.); 2 Department of Pharmaceutical Engineering and Biotechnology, Sun Moon University, Asan 31460, Republic of Korea; llee5405@gmail.com; 3 Genome-Based BioIT Convergence Institute, Sun Moon University, Asan 31460, Republic of Korea

**Keywords:** non-small cell lung cancer, cancer stem cell, cyclophilin A, CD147, epidermal growth factor receptor, compound 9, cyclosporin A, afatinib

## Abstract

Non-small cell lung cancer (NSCLC) is a fatal malignant tumor with a high mortality rate. Cancer stem cells (CSCs) play pivotal roles in tumor initiation and progression, treatment resistance, and NSCLC recurrence. Therefore, the development of novel therapeutic targets and anticancer drugs that effectively block CSC growth may improve treatment outcomes in patients with NSCLC. In this study, we evaluated, for the first time, the effects of natural cyclophilin A (CypA) inhibitors, including 23-demethyl 8,13-deoxynargenicin (C9) and cyclosporin A (CsA), on the growth of NSCLC CSCs. C9 and CsA more sensitively inhibited the proliferation of epidermal growth factor receptor (EGFR)-mutant NSCLC CSCs than EGFR wild-type NSCLC CSCs. Both compounds suppressed the self-renewal ability of NSCLC CSCs and NSCLC-CSC-derived tumor growth in vivo. Furthermore, C9 and CsA inhibited NSCLC CSC growth by activating the intrinsic apoptotic pathway. Notably, C9 and CsA reduced the expression levels of major CSC markers, including integrin α6, CD133, CD44, ALDH1A1, Nanog, Oct4, and Sox2, through dual downregulation of the CypA/CD147 axis and EGFR activity in NSCLC CSCs. Our results also show that the EGFR tyrosine kinase inhibitor afatinib inactivated EGFR and decreased the expression levels of CypA and CD147 in NSCLC CSCs, suggesting close crosstalk between the CypA/CD147 and EGFR pathways in regulating NSCLC CSC growth. In addition, combined treatment with afatinib and C9 or CsA more potently inhibited the growth of EGFR-mutant NSCLC CSCs than single-compound treatments. These findings suggest that the natural CypA inhibitors C9 and CsA are potential anticancer agents that suppress the growth of EGFR-mutant NSCLC CSCs, either as monotherapy or in combination with afatinib, by interfering with the crosstalk between CypA/CD147 and EGFR.

## 1. Introduction

Non-small cell lung cancer (NSCLC), which accounts for approximately 85% of all lung cancer cases, is one of the most commonly diagnosed cancers and a leading cause of cancer-related deaths worldwide [1,2]. Recent advances in NSCLC treatment have enabled surgical resection, radiotherapy, and customized targeted therapies [3]. Although epidermal growth factor receptor tyrosine kinase inhibitors (EGFR TKIs), such as erlotinib, gefitinib, and afatinib, provide favorable treatment outcomes in EGFR-mutation-positive patients, the overall survival rate of NSCLC patients remains low, and most NSCLC patients experience cancer recurrence and metastasis [4,5,6,7]. Therefore, exploring new targets and developing new drugs are critical for improving the current therapy and survival rates of patients with NSCLC.

Numerous studies have demonstrated that lung cancer is a highly heterogeneous tumor and that cancer stem cells (CSCs) underlie the malignant development, progression, invasion, metastasis, and recurrence of NSCLC [8,9]. NSCLC CSCs express specific stemness markers, including integrin α6, CD133, CD44, Nanog, Oct4, aldehyde dehydrogenase 1 (ALDH1), and Sox2, which are positively associated with aggressive biological behavior and poor prognosis in NSCLC [9,10,11]. Therefore, new drugs that inhibit these lung-cancer-specific stemness markers may be an effective cancer treatment strategy for inhibiting NSCLC CSCs and overcoming drug resistance.

Cyclophilin A (CypA) is the most abundant cyclophilin with peptidyl prolyl isomerase (PPIase) activity. It catalyzes the isomerization of peptide bonds from *trans-* to *cis-*form at proline residues [12,13]. CypA regulates protein folding and trafficking, immune cell activation, and cell signaling [14]. It is an intracellular protein that is secreted extracellularly in response to inflammatory stimuli [15]. CD147 (also known as basigin or EMMPRIN), a transmembrane glycoprotein belonging to the immunoglobulin superfamily, functions as a major signaling receptor for extracellular CypA [16]. CypA/CD147 interactions contribute to the development of several human diseases, including cancer [14]. Activation of the CypA/CD147 axis plays an important role in cancer cell proliferation, metastasis, antiapoptosis, chemotherapy resistance, and radiotherapy resistance, as well as CSC initiation, growth, and survival [14]. Thus, CypA/CD147 is a potential target for eradicating cancer cells and CSCs.

**Figure 1:**
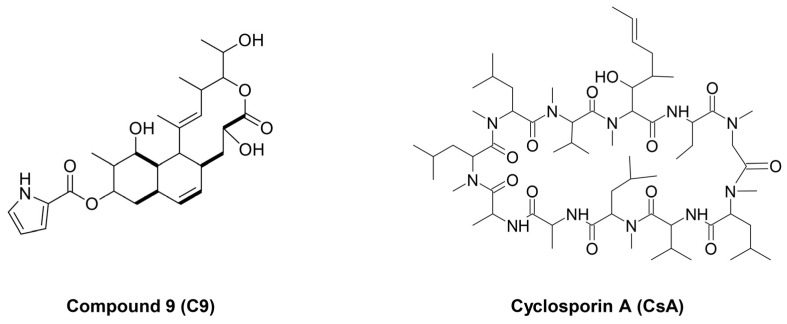
The chemical structures of C9 and CsA.

CypA was first identified as a target protein of the immunosuppressive agent cyclosporin A (CsA), which interferes with PPIase activity [17]. The fungal metabolite, CsA, is an extracellular and intracellular CypA inhibitor with a wide range of biological activities, including immunosuppressive, anti-inflammatory, antifungal, and antitumor effects [17] (Figure 1). CsA inhibits CypA/CD147-mediated downstream signaling and cellular functions by interfering with the binding of CypA to CD147 [18]. Moreover, CsA promotes gefitinib-induced apoptosis by inhibiting the signal transducer and activator of the transcription 3 (STAT3) pathway in EGFR-TKI-sensitive and -resistant NSCLC cells [19].

In our previous study, 23-demethyl 8,13-deoxynargenicin (compound 9; C9), a novel analog of the antibacterial macrolide nargenicin A1, a major secondary metabolite produced by the *Nocardia* species, was identified as a new CypA inhibitor [20] (Figure 1). C9 exhibits potential anticancer activity by targeting the CypA/CD147 interaction [21]. C9 downregulates CD147-mediated mitogen-activated protein kinase (MAPK) signaling pathways, including c-Jun N-terminal kinase (JNK) and extracellular signal-regulated protein kinase 1/2 (ERK1/2), by suppressing CypA and CD147 expression in gastric cancer (GC) cells [21]. C9 inhibits the proliferation, migration, and invasion of GC cells. Moreover, C9 suppresses GC-cell-induced angiogenesis by downregulating vascular endothelial growth factor (VEGF)/VEGF receptor 2 signaling and the hypoxia-inducible factor 1-alpha/VEGF pathway [22]. Recently, we demonstrated the therapeutic potential of C9 and CsA in effectively suppressing gastric cancer stem cell (GCSC) propagation by targeting the CypA/CD147 axis [23]. Natural CypA inhibitors suppress the proliferation and induce the apoptosis of GCSCs by regulating the CypA/CD147-mediated protein kinase B (AKT) and MAPK signaling pathways. However, whether the CypA inhibitors C9 and CsA inhibit NSCLC CSCs, which are key targets of lung cancer, and the underlying mechanisms remains unexplored.

In the present study, we demonstrated for the first time the significant anticancer effects of natural CypA inhibitors on NSCLC CSCs in vitro and in vivo. In addition, our results suggest that the anticancer activities of C9 and CsA in NSCLC CSCs may be associated with the blockade of the crosstalk between CypA/CD147 and EGFR.

## 2. Results

### 2.1. C9 and CsA Suppress the Growth of NSCLC CSCs

CSCs were enriched from NSCLC cell lines as tumorspheres under CSC-selective culture conditions in a serum-free medium [24,25]. To investigate whether C9 and CsA affect the proliferation of NSCLC CSCs, CSCs derived from four EGFR-wild-type (A549 and NCI-H1299) or -mutant (NCI-H1650 and HCC827) NSCLC cell lines were treated with C9 or CsA at various concentrations (0–100 µM) for 4 days. Cell proliferation was evaluated using a luminescent ATP detection assay. As shown in Table 1, C9 inhibited the proliferation of A549-, NCI-H1299-, NCI-H1650-, and HCC827-derived NSCLC CSCs with IC_50_ values of 49.03, 57.56, 29.18, and 25.57 µM, respectively. The IC_50_ values of CsA in the four NSCLC CSCs were 9.34, 9.72, 2.47, and 8.90 µM, respectively. These data indicate that natural CypA inhibitors more potently inhibited the proliferation of EGFR-mutant NSCLC CSCs than EGFR-wild-type NSCLC CSCs. Based on these results, we further assessed the anticancer effects of C9 and CsA on NSCLC CSCs derived from the EGFR-mutant cells, NCI-H1650 and HCC827, which were more sensitive to C9 and CsA.

**Figure 2:**
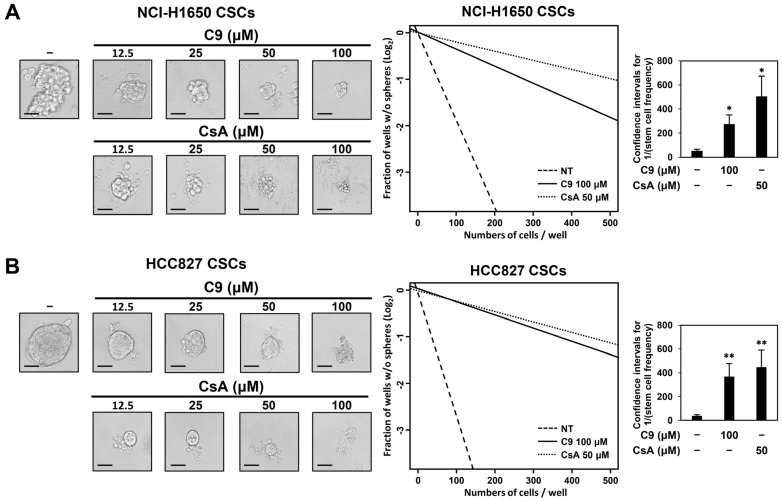
Effects of C9 and CsA on the self-renewal of NSCLC CSCs using limiting dilution assay (LDA). (**A**) NCI-H1650- and (**B**) HCC827-derived NSCLC CSCs were plated with limiting dilutions (from 5 to 500 cells/well) using serum-free media. The cells were then treated with the indicated concentrations of C9 or CsA for 4 days. Formed tumorspheres were observed under an optical microscope, and the number of wells containing spheres were quantified. The frequency of tumorsphere formation (stem cell frequency) was measured by extreme limiting dilution analysis (ELDA) software (http://bioinf.wehi.edu.au/software/elda/) (accessed on 15 August 2021). * *p* < 0.05, ** *p* < 0.01 vs. the control group. Scale bars: 50 µm.

Self-renewal is essential for CSC maintenance and growth [26]. To evaluate the effects of C9 and CsA on the self-renewal capacity of NSCLC CSCs, we performed a limiting dilution assay (LDA), a widely used method to measure stem cell frequency [27,28]. An extremely low number of diluted cells per well were plated, and the presence and number of tumorspheres in each well were quantified. As shown in Figure 2, C9 and CsA markedly inhibited the formation and growth of tumorspheres in both NCI-H1650- and HCC827-derived NSCLC CSCs in a concentration-dependent manner. The C9- and CsA-treated NCI-H1650 CSCs showed five- and nine-fold lower tumorsphere-forming frequencies, respectively, than untreated control cells at the indicated concentrations of the compounds (Figure 2A). Treatment with C9 and CsA also led to nine- and eleven-fold lower tumorsphere-forming frequencies, respectively, in HCC827 CSCs (Figure 2B). These results demonstrate that CypA inhibitors effectively suppress the self-renewal of NSCLC CSCs.

### 2.2. C9 and CsA Suppress NSCLC CSC-Derived Tumor Growth In Vivo

**Figure 3:**
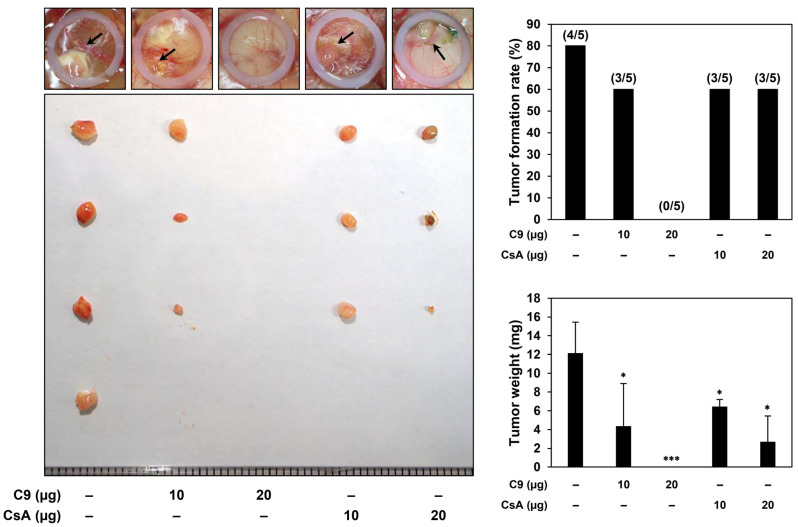
Effects of C9 and CsA on the NSCLC CSC-derived tumor growth in a CAM model. NCI-H1650-derived CSCs were mixed with ECM gel in the absence or presence of C9 or CsA (10 or 20 μg/egg) and implanted onto the CAM surface of fertilized chick eggs (5 eggs per group). After 9 days of incubation, the CAMs were observed, the formed tumors were recovered, and the tumor formation rate and tumor weight were analyzed. * *p* < 0.05, *** *p* < 0.001 vs. the control group.

To further confirm the effects of C9 and CsA on the tumorigenic potential of NSCLC CSCs in vivo, a chicken embryonic chorioallantoic membrane (CAM) tumor model transplanted with NCI-H1650-derived CSCs was used. As shown in Figure 3, the tumor formation rate of the untreated control group was 80% (tumor formation in 4 out of 5 eggs), whereas the tumor formation rate of the C9 (10 μg/egg)- or CsA (10 or 20 μg/egg)-treated groups was 60% (tumor formation in 3 out of 5 eggs). Notably, the C9 treatment (20 µg/egg) completely inhibited tumor formation (no tumor formation in all 5 eggs). In addition, the tumor weight of the control group was 12.8 ± 3.7 mg, whereas the tumor weight after C9 (10 μg/egg) or CsA (10 or 20 μg/egg) treatment was 4.4 ± 4.5, 6.4 ± 0.8, and 2.7 ± 2.7 mg, respectively. These data indicate that C9 and CsA significantly inhibit NSCLC-CSC-derived tumor growth in vivo. In particular, C9 showed a stronger growth-inhibitory effect than CsA on NSCLC CSCs in the CAM tumor model, suggesting that C9 may have greater therapeutic potential than CsA to eradicate NSCLC CSCs in vivo.

### 2.3. C9 and CsA Promote Apoptosis in NSCLC CSCs

**Figure 4:**
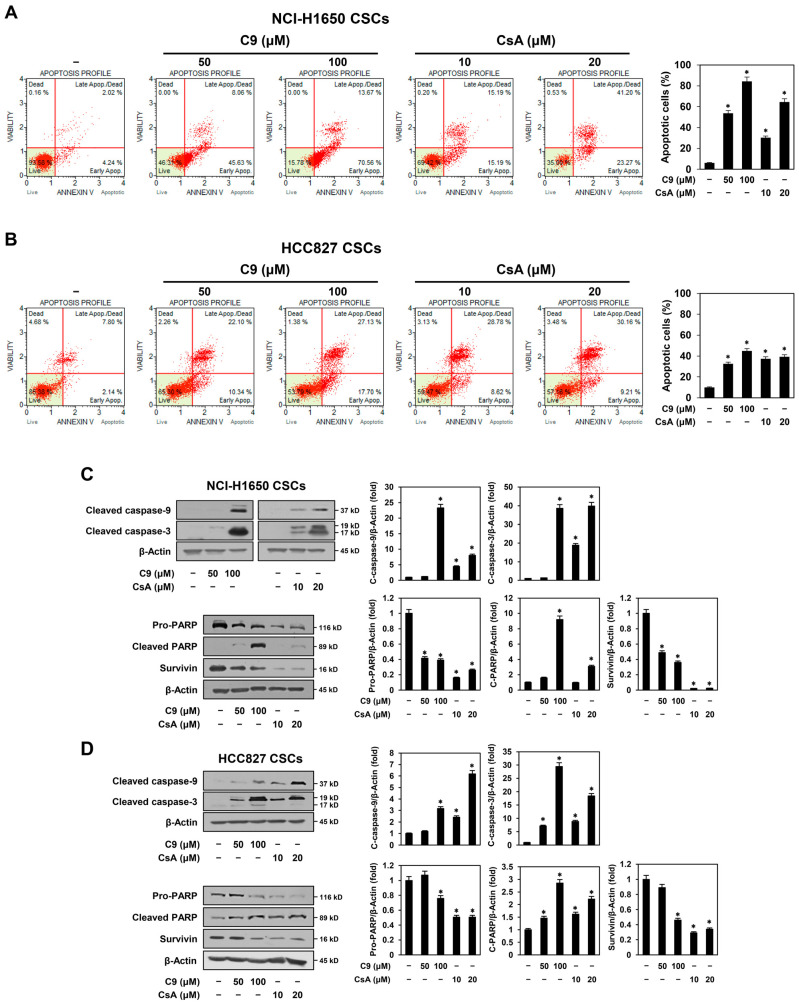
Effects of C9 and CsA on the apoptosis of NSCLC CSCs. NCI-H1650- and HCC827-derived CSCs were treated with the indicated concentrations of C9 or CsA for 48 h. (**A**,**B**) Apoptotic cells were detected using a Muse Cell Analyzer with Muse^®^ Annexin V & Dead Cell Kit. (**C**,**D**) Protein levels of apoptosis regulators were detected by Western blot analysis using specific antibodies and were further quantified by densitometry. β-Actin levels were used as an internal control. * *p* < 0.05 vs. the control group.

CSCs exhibit increased resistance to apoptosis induction [29]. To determine whether C9 and CsA promote the apoptosis of NSCLC CSCs, the apoptosis of NCI-H1650- and HCC827-derived CSCs treated with C9 or CsA was analyzed by flow cytometry. As shown in Figure 4A,B, C9 and CsA significantly increased the proportion of apoptotic cells compared to the untreated control group in both NSCLC CSCs. These results indicate that the inhibitory effects of C9 and CsA on NSCLC CSC growth are related to an increase in apoptosis.

Next, we assessed whether C9 and CsA regulate the apoptotic pathway in NSCLC CSCs. The apoptotic response is activated through either the intrinsic or extrinsic pathway [30]. Activated caspase-9 in the intrinsic pathway triggers the cleavage and activation of caspase-3. Subsequently, caspase-3 cleaves poly(ADP-ribose)polymerase (PARP), a DNA repair enzyme, and thus promotes apoptosis [30]. As shown in Figure 4C,D, treatment with C9 or CsA increased the expression of cleaved caspase-9 and cleaved caspase-3 in NCI-H1650- and HCC827-derived CSCs. Furthermore, the expression levels of pro-PARP were decreased by C9 or CsA treatment, whereas those of cleaved PARP were increased in both NSCLC CSCs. In addition, C9 and CsA effectively suppressed the expression of survivin, a member of the inhibitor of the apoptosis protein (IAP) family, which interferes with the activity of caspases, such as caspase-3, -7, and -9, in NCI-H1650- and HCC827-derived CSCs [31] (Figure 4C,D). Taken together, these results suggest that natural CypA inhibitors may inhibit NSCLC CSC growth by activating the intrinsic apoptotic pathway.

### 2.4. C9 and CsA Inhibit CSC Biomarker Expression through Dual Downregulation of CypA/CD147 and EGFR in NSCLC CSCs

**Figure 5:**
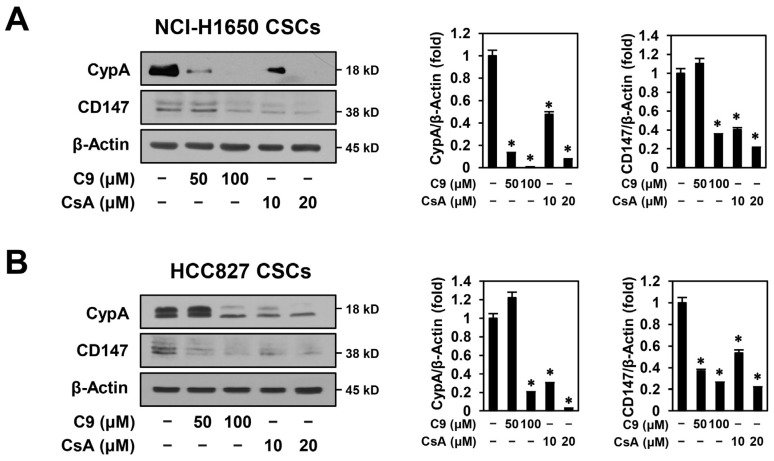

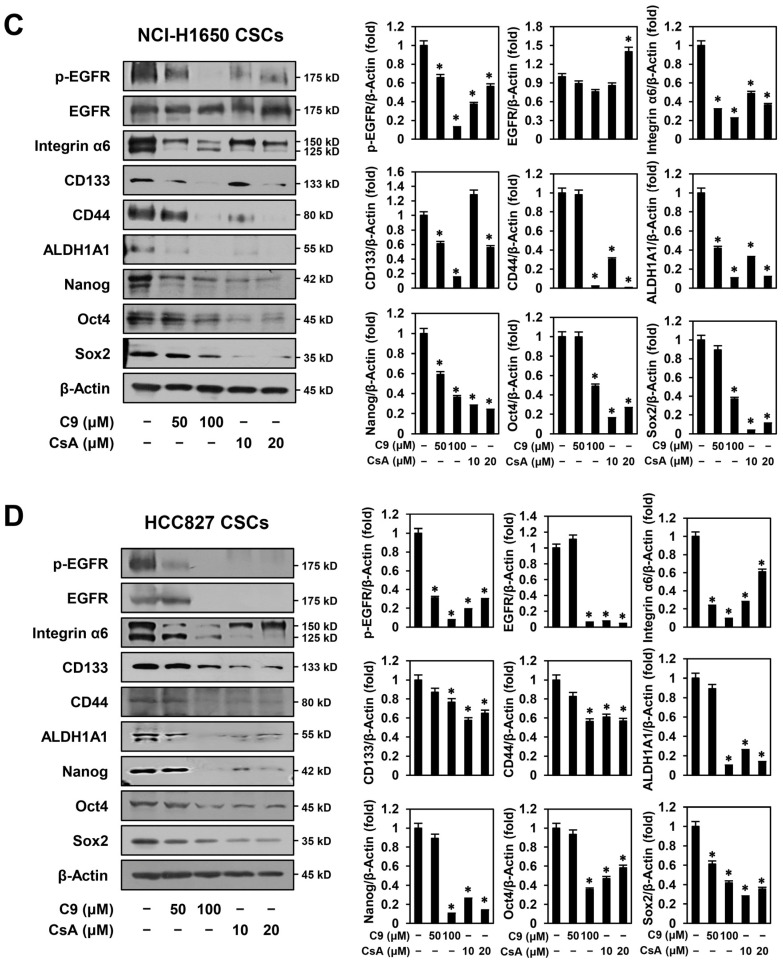
Effects of C9 and CsA on the expression of CypA, CD147, EGFR, and CSC markers in NSCLC CSCs. (**A**–**D**) NCI-H1650- and HCC827-derived CSCs were treated with the indicated concentrations of C9 or CsA for 48 h. Protein levels were detected by Western blot analysis using specific antibodies and were further quantified by densitometry. β-Actin levels were used as an internal control. * *p* < 0.05 vs. the control group.

The CypA/CD147 axis plays a crucial role in CSC initiation, growth, maintenance, and metastasis [14]. Thus, we investigated whether C9 and CsA affected the expression of CypA and CD147 in NSCLC CSCs. As shown in Figure 5A,B, the expression levels of CypA and CD147 were reduced by treatment with C9 or CsA in a dose-dependent manner in NCI-H1650- and HCC827-derived CSCs, implying that the anticancer activities of C9 and CsA on NSCLC CSCs resulted from the downregulation of the CypA/CD147 axis.

Dysfunctional EGFR signaling promotes the acquisition of cancer stem-like properties, including self-renewal, tumor heterogeneity, metastasis, resistance to chemotherapy and radiotherapy, and recurrence, by increasing the expression of CSC-related markers in NSCLC [32,33,34]. Therefore, the inhibition of CSC markers may be an effective strategy to eliminate NSCLC CSCs. The major stem cell markers for lung cancer include surface biomarkers, such as CD44, CD133, and integrin α6, and intracellular biomarkers, such as ALDH1, Nanog, Oct4, and Sox2 [32,33,34]. Therefore, we investigated the effects of C9 and CsA on the expression of EGFR and CSC markers. Both C9 and CsA significantly inhibited EGFR activity in NCI-H1650- and HCC827-derived CSCs (Figure 5C,D). These two compounds effectively inhibited EGFR phosphorylation in both NSCLC CSCs. However, they did not affect total EGFR expression levels in NCI-H1650-derived CSCs, but reduced them in HCC827-derived CSCs. Similar to these effects of CypA inhibitors, CypA knockdown using the CypA-specific siRNA reduced EGFR phosphorylation in both NSCLC CSCs (Figure S1). Furthermore, C9 and CsA potently suppressed the expression of integrin α6, CD133, CD44, ALDH1A1, Nanog, Oct4, and Sox2 in both NSCLC CSCs (Figure 5C,D). Taken together, these data suggest that natural CypA inhibitors suppress the expression of CSC markers through dual downregulation of the CypA/CD147 axis and EGFR activity in NSCLC CSCs.

### 2.5. Afatinib Inhibits CypA/CD147 Expression in NSCLC CSCs

**Figure 6:**
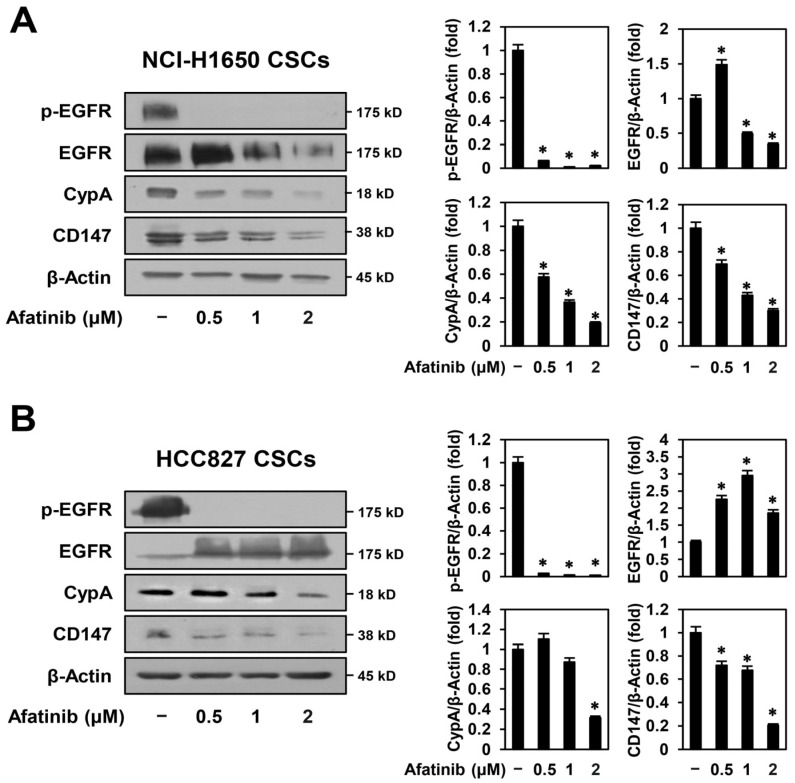
Effect of afatinib on the expression of EGFR, CypA, and CD147 in NSCLC CSCs. (**A**) NCI-H1650- and (**B**) HCC827-derived CSCs were treated with the indicated concentrations of afatinib for 48 h. Protein levels were detected by Western blot analysis using specific antibodies and were further quantified by densitometry. β-Actin levels were used as an internal control. * *p* < 0.05 vs. the control group.

To confirm the mechanistic association between CypA/CD147 and EGFR in NSCLC CSCs, we assessed whether treatment with afatinib, a second-generation EGFR TKI, affected the expression levels of CypA and CD147 in NCI-H1650- and HCC827-derived CSCs. As shown in Figure 6A,B, afatinib not only remarkably suppressed EGFR phosphorylation but also effectively reduced the expression levels of CypA and CD147 in both NSCLC CSCs. Our findings that the CypA inhibitors C9 and CsA inhibit EGFR activity and the EGFR inhibitor afatinib inhibits CypA/CD147 expression suggest that there is close crosstalk between the CypA/CD147 and EGFR pathways in regulating the stem-like properties of NSCLC. In conclusion, C9 and CsA may suppress NSCLC CSC growth by interfering with the crosstalk between the CypA/CD147 and EGFR pathways.

### 2.6. Combined Treatment with CypA Inhibitors and Afatinib More Potently Inhibits the Growth of EGFR-Mutated NSCLC CSCs than Single-Compound Treatments

**Figure 7:**
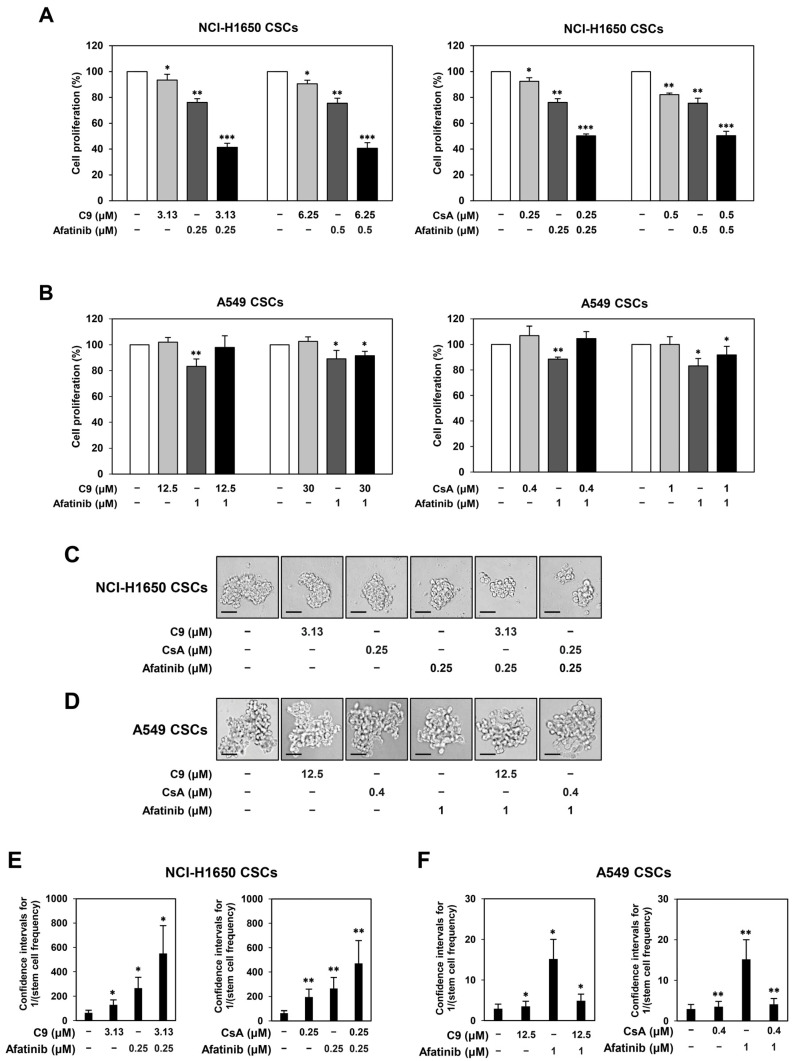
Effects of combined treatment with CypA inhibitors and afatinib on the growth of NSCLC CSCs. NCI-H1650- and A549-derived CSCs were treated with the indicated concentrations of C9, CsA, or Afatinib for 4 days. (**A**,**B**) Cell proliferation was measured using the CellTiter-Glo^®^ luminescent assay system. (**C**,**D**) Formed tumorspheres were observed under an optical microscope. (**E**,**F**) The number of wells containing spheres were quantified. The frequency of tumorsphere formation (stem cell frequency) was measured by extreme limiting dilution analysis (ELDA) software (http://bioinf.wehi.edu.au/software/elda/) (accessed on 18 October 2021). * *p* < 0.05, ** *p* < 0.01, *** *p* < 0.001 vs. the control group. Scale bars: 50 µm.

Although afatinib is one of the standard treatments for patients with EGFR-mutated NSCLC, it is necessary to develop a combination therapy to overcome poor treatment outcomes, such as drug resistance and tumor recurrence [7]. Based on the possible crosstalk between CypA/CD147 and EGFR, we assessed the effects of the combined treatment with CypA inhibitors and afatinib on the growth of NSCLC-derived CSCs using concentrations below the IC_30_ value of each compound. As shown in Figure 7A, cotreatment with afatinib and C9 or CsA more potently inhibited the proliferation of EGFR-mutant NCI-H1650-derived CSCs compared to single-compound treatments. However, the combination treatment did not inhibit the proliferation of EGFR-wild-type A549-derived CSCs (Figure 7B). In addition, combined treatment with afatinib and C9 or CsA suppressed the tumorsphere formation ability of NCI-H1650-derived CSCs more effectively than single-compound treatment (Figure 7C,E), but did not suppress the tumorsphere formation ability of A549-derived CSCs (Figure 7D,F). These findings suggest that the natural CypA inhibitors, C9 and CsA, can be used in combination with afatinib to treat NSCLC harboring EGFR mutations by targeting CSCs. Notably, cotreatment with afatinib and C9 or CsA more potently reduced the expression levels of CypA and CD147 in NCI-H1650-derived CSCs in comparison with the treatment of each compound alone (Figure S2). In addition, the phosphorylation of EGFR was remarkably suppressed by the combination treatment in NCI-H1650-derived CSCs (Figure S2). Therefore, the combination effect of afatinib with C9 or CsA in EGFR-mutant NSCLC CSCs may be related to a stronger blockade of the CypA/CD147/EGFR axis.

## 3. Discussion

EGFR plays a central role in regulating cell proliferation and is the most common oncogenic driver in NSCLC [35]. Mutations in the gene encoding EGFR result in the overexpression or increased tyrosine kinase activity of the protein. These EGFR aberrations promote cancer cell proliferation by activating downstream oncogenic signaling pathways [36]. Most patients with advanced NSCLC have EGFR mutations and receive first-line standard treatment with EGFR TKIs, such as erlotinib, gefitinib, icotinib, afatinib, dacomitinib, and osimertinib [7]. However, treatment failure with primary EGFR TKIs leads to drug resistance and metastasis, resulting in low survival rates in patients with NSCLC [7]. CSCs are a major cause of treatment failure and poor prognosis in patients with lung cancer owing to drug resistance, metastasis, and recurrence [32]. EGFR TKI treatment increased the population of CSCs in EGFR-mutated NSCLC, and CSC-related markers, such as CD44, CD133, integrin α6, Oct4, Sox2, Nanog, and ALDH1, were highly expressed in patients with EGFR-TKI-resistant NSCLC [33,34]. The cell surface markers CD44, CD133, and integrin α6 play crucial roles in the growth, self-renewal, metastasis, and chemoresistance of NSCLC CSCs by regulating several signaling pathways, including the Notch, Hedgehog, Wnt, and STAT3 pathways [32,36,37,38]. ALDH1, an important enzyme in the detoxification of endogenous and exogenous aldehyde substrates through NAD(P)^+^-dependent oxidation, is important for the maintenance and differentiation of CSCs and is overexpressed in NSCLC, resulting in increased treatment resistance [39]. The intracellular transcription factors Sox2, Nanog, and Oct4 activate the expression of stemness-related genes to promote the development and recurrence of NSCLC [40,41,42]. Therefore, there is an urgent need to explore potential molecular targets and novel chemotherapeutic agents that can inhibit lung-cancer-specific stemness markers to improve NSCLC treatment outcomes and reduce mortality.

Numerous studies have demonstrated that CypA and its receptor CD147 are overexpressed in many cancers, including lung cancer, and that the activation of CypA/CD147-mediated intracellular downstream signaling pathways promotes the growth, metastasis, therapeutic resistance, and stem-like properties of cancer cells [14]. In addition, a significant association has been observed between CypA/CD147 overexpression and the poor prognosis of patients with cancer, such as a low survival rates and advanced cancer stages [14]. Notably, the CypA/CD147 axis plays an important role in the initiation, growth, and survival of CSCs by upregulating major signaling pathways, including Wnt/β-catenin, STAT3, Notch, phosphatidylinositol 3-kinase (PI3K)/AKT, MAPK, and nuclear factor kappa B (NF-κB), in several cancers, such as glioma, colon cancer, pancreatic cancer, and breast cancer [13,41,42,43,44]. Therefore, the CypA/CD147 axis is an attractive target for CSC eradication. However, the role of the CypA/CD147 axis in the maintenance of NSCLC CSCs is still unclear.

To evaluate the potential of the CypA/CD147 axis as a therapeutic target for NSCLC CSC elimination, we investigated the anticancer effects and underlying molecular mechanisms of the natural CypA inhibitors, C9 and CsA, on the growth of NSCLC CSCs in the present study. Our results show that C9 and CsA inhibited the proliferation of EGFR-mutant NSCLC CSCs more sensitively than EGFR-wild-type NSCLC CSCs, indicating that CypA inhibitors exhibit more effective antiproliferative activity against CSCs derived from NSCLC cells with increased EGFR activity. Moreover, C9 and CsA significantly suppressed the self-renewal ability of EGFR-mutant NSCLC CSCs in vitro and their tumorigenic potential in vivo. Overexpression of survivin, a member of the IAP family that inhibits caspases and blocks apoptosis, is associated with the aberrant activation of EGFR and is positively correlated with poor prognosis in patients with NSCLC [43,44]. C9 and CsA lead to apoptosis in EGFR-mutant NSCLC CSCs through activation of the intrinsic apoptotic pathway by reducing survivin expression levels. Recent studies have revealed that dysfunctional EGFR signaling promotes the acquisition of cancer stem-like traits by increasing the expression levels of cancer stemness markers in NSCLC [32,33,34]. Our results show that C9 and CsA potently suppressed the expression of the CSC markers, including integrin α6, CD133, CD44, ALDH1A1, Nanog, Oct4, and Sox2, by downregulating CypA/CD147 expression and EGFR activity in EGFR-mutant NSCLC CSCs. Furthermore, we confirmed that the EGFR TKI, afatinib, significantly suppressed the expression of CypA and CD147 in EGFR-mutant NSCLC CSCs, suggesting that there may be close crosstalk between the CypA/CD147 and EGFR pathways in maintaining the stem-like properties of NSCLC. There is accumulating evidence demonstrating the interaction between CypA/CD147 and EGFR in the regulation of various cellular functions [45,46,47,48]. Cyp obtained from red algae stimulates intestinal epithelial cell proliferation via the EGFR signaling pathway [45]. CD147 forms a complex with EGFR and induces breast epithelial cell invasiveness by activating the EGFR/Ras/ERK signaling pathway [46]. In addition, CD147 promotes pancreatic cancer cell invasion by upregulating the EGFR/STAT3 signaling pathway [47]. Membrane localization of EGFR was higher in cells with high levels of CD147 expression than in cells with low levels of CD147 expression [48]. Collectively, these findings indicate that C9 and CsA may suppress the growth of EGFR-mutant NSCLC CSCs by interfering with the crosstalk between the CypA/CD147 and EGFR pathways. However, we need to further identify the detailed molecular mechanisms regulating the functional interplay of the two pathways. In addition, to further clarify the effects of the CypA inhibitors and the mode of EGFR downregulation in EGFR-mutant NSCLC CSCs, it is necessary to evaluate the anticancer activities of C9 and CsA using CSCs derived from different types of EGFR-mutant NSCLC cell lines.

Combination therapy is a strategic approach for overcoming chemotherapy resistance, side effects, and treatment failure [49]. Cotreatment with afatinib and bevacizumab, an antiangiogenic monoclonal antibody, inhibited tumor growth in patients with EGFR-mutant NSCLC, without side effects [49]. The combination of PI-103, a dual PI3K and mTOR inhibitor, and afatinib synergistically inhibited the growth of NSCLC cells [50]. In addition, afatinib inhibited NSCLC cell proliferation by acting synergistically with selumetinib and trametinib, which are mitogen-activated protein kinase kinase (MEK) inhibitors [51]. However, the combined effects of CypA and EGFR inhibitors have not been evaluated. Our results show that the cotreatment with afatinib and the CypA inhibitors, C9 or CsA, more potently inhibited the proliferation and tumorsphere formation of EGFR-mutant NSCLC CSCs than single-compound treatments, but did not suppress these processes in EGFR-wild-type NSCLC CSCs. These results suggest that the simultaneous targeting of CypA/CD147 and EGFR can more effectively suppress EGFR-mutant NSCLC CSC growth by disrupting crosstalk between the CypA/CD147 and EGFR pathways. In particular, the potent combination effects were shown at much lower concentrations of the CypA inhibitors than the effective dosages of each single agent, indicating that C9 and CsA may have stronger therapeutic potential in combination therapy with afatinib than in monotherapy.

Interestingly, the CypA inhibitors C9 and CsA, either alone or in combination with afatinib, were more effective against EGFR-mutant NSCLC CSCs than EGFR-wild-type NSCLC CSCs. EGFR-mutant NSCLC cells with increased EGFR tyrosine kinase activity excessively activate multiple EGFR-mediated downstream signaling pathways, such as MAPK, PI3K/AKT, and STAT3 pathways, consequently promoting the overexpression of CSC biomarkers and enhancing the stem-like properties of the cells [32,33,34,52]. Therefore, C9 and CsA may more potently suppress the growth of EGFR-mutant NSCLC CSCs with higher EGFR dependence than EGFR-wild-type NSCLC CSCs by targeting the CypA/CD147 axis. Nevertheless, further studies are needed to understand the molecular mechanisms responsible for the enhanced anticancer activity of CypA inhibitors against EGFR-mutant NSCLC CSCs. Additionally, in vivo animal model studies are required to comprehensively evaluate the efficacy and safety of CypA inhibitors as potential anticancer agents for the treatment of NSCLC. Taken together, our findings suggest that the CypA/CD147 axis is a potent therapeutic target for EGFR-mutant NSCLC CSCs and that the natural CypA inhibitors C9 and CsA may serve as novel therapeutic agents to eliminate EGFR-mutant NSCLC CSCs, either as monotherapy or in combination with afatinib.

## 4. Materials and Methods

### 4.1. Materials

C9 was isolated from the culture extract of *Nocardia* sp. CS682 mutant as previously described [20]. CsA and afatinib were purchased from Sigma-Aldrich (St. Louis, MO, USA). The compounds were prepared at a concentration of 100 mM using dimethyl sulfoxide (DMSO). DMEM/F12 and Accutase were purchased from HyClone (Marlborough, MA, USA) and EMD Millipore (Temecula, CA, USA), respectively. Basic fibroblast growth factor (bFGF) and epidermal growth factor (EGF) were purchased from Prospecbio (East Brunswick, NJ, USA). B-27 serum-free supplement, L-glutamine, and penicillin/streptomycin were purchased from Gibco (Grand Island, NY, USA). Extracellular matrix (ECM) gel from Engelbreth-Holm-Swarm murine sarcoma and heparin were purchased from Sigma-Aldrich (St. Louis, MO, USA). The CellTiter-Glo^®^ 2.0 Cell Viability Assay and Muse^®^ Annexin V & Dead Cell Kits were purchased from Promega (Madison, WI, USA) and Luminex (Austin, TX, USA), respectively. Antibodies against cleaved caspase-9 (cat. no. 9501), cleaved caspase-3 (cat. no. 9661), survivin (cat. no. 2808), PARP (cat. no. 9542), CypA (cat. no. 2175), CD147 (cat. no. 13287), phospho-EGFR (Tyr1068, cat. no. 2234), EGFR (cat. no. 2232), integrin α6 (cat. no. 3750), CD133 (cat. no. 64326), CD44 (cat. no. 37259), ALDH1A1 (cat. no. 12035), Nanog (cat. no. 3580), Oct4 (cat. no. 2750), Sox2 (cat. no. 3579), β-actin (cat. no. 4967), rabbit immunoglobulin G (IgG) (cat. no. 7074), and mouse IgG (cat. no. 7076) were purchased from Cell Signaling Technology (Danvers, MA, USA).

### 4.2. NSCLC CSC Culture

Human NSCLC cell lines A549 (KCLB No. 10185), NCI-H1299 (KCLB No. 25803), NCI-H1650 (KCLB No. 91650), and HCC827 (KCLB No. 70827) were obtained from the Korean Cell Line Bank (Seoul, Republic of Korea). The identity of each cell line was confirmed by STR profiling: A549 (D3S1358: 16; vWA: 14; FGA: 23; Amelogenin: X,Y; TH01: 8,9.3; TPOX: 8,11; CSF1PO: 10,12; D5S818: 11; D13S317: 11; D7S820: 8,11), NCI-H1299 (D3S1358: 17; vWA: 16,18; FGA: 20; Amelogenin: X; TH01: 6,9.3; TPOX: 8; CSF1PO: 12; D5S818: 11; D13S317: 12; D7S820: 10), NCI-H1650 (D3S1358: 18; vWA: 18; FGA: 20,23.2; Amelogenin: X; TH01: 9.3; TPOX: 11; CSF1PO: 11; D5S818: 11; D13S317: 11; D7S820: 8,9), and HCC827 (D3S1358: OL; vWA: 18; FGA: 22,24; Amelogenin: X; TH01: 6; TPOX: 8; CSF1PO: 11; D5S818: 12; D13S317: 9; D7S820: 11,12). CSCs were propagated from NSCLC cell lines as tumorspheres under CSC-selective culture conditions, and their stem properties were confirmed as previously described [23,24,25]. NSCLC tumorsphere cells were cultured in serum-free DMEM/F12 containing 20 ng/mL EGF, 20 ng/mL bFGF, 1 × B-27, 5 μg/mL heparin, 2 mM L-glutamine, and 1% penicillin/streptomycin. Tumorspheres were subcultured through dissociation with Accutase. The cells were maintained at 37 °C in a humidified CO_2_ incubator with 5% CO_2_ (Thermo Scientific, Vantaa, Finland).

### 4.3. Cell Proliferation Assay

Cell proliferation was quantitatively measured using the CellTiter-Glo^®^ 2.0 Cell Viability Assay Kit as previously described [53]. Briefly, NSCLC-derived CSCs (3 × 10^3^ cells/well) were seeded in a 96-white-well culture plate using serum-free media and treated with different concentrations of each compound for 4 days. The luminescent signal was detected using a multimode microplate reader (BioTek, Inc., Winooski, VT, USA). The IC_50_ values were calculated using GraphPad Prism 6 (GraphPad Software, La Jolla, CA, USA).

### 4.4. Limiting Dilution Assay

The tumorsphere-forming ability of NSCLC CSCs was analyzed by in vitro extreme limiting dilution analysis (ELDA). NCI-H1650- and HCC827-derived CSCs dissociated into single-cell were seeded in a 96-well culture plate with various seeding densities (5–500 cells/well) using serum-free media and treated with the indicated concentrations of each compound for 4 days. The presence and number of tumorspheres in each well were analyzed under an optical microscope (Olympus, Tokyo, Japan). The frequency of tumorsphere formation (stem cell frequency) was measured by ELDA software (http://bioinf.wehi.edu.au/software/elda/).

### 4.5. Chick Embryo Chorioallantoic Membrane (CAM) Assay

To evaluate the effects of the compounds on the tumorigenic potential of NSCLC CSCs in vivo, a CAM assay was performed as previously described [53]. Briefly, fertilized chick eggs were incubated at 37 °C in a humidified egg incubator for 7 days, and the eggshell membrane was carefully peeled away. NCI-H1650-derived CSCs (2.5 × 10^6^ cells/egg) were mixed with ECM gel (40 μL/egg) in the absence or presence of the compounds (10 or 20 μg/egg) and placed onto the CAM inside the silicone ring (5 eggs per group). After incubation for 9 days, the tumor formed on the CAM was retrieved, and the formation rate and weight of the tumor were measured.

### 4.6. Analysis of Apoptosis

NCI-H1650- and HCC827-derived CSCs (1 × 10^5^ cells/well) were placed in a 12-well culture plate using serum-free media and treated with the indicated concentrations of each compound for 48 h. The cells were collected and stained with Muse^®^ Annexin V & Dead Cell reagent as previously described [53]. The percentage of apoptotic cells were analyzed using a Guava^®^ Muse^®^ Cell Analyzer (MuseSoft_V1.8.0.3; Luminex Corporation, Austin, TX, USA).

### 4.7. Western Blot Analysis

Cell lysates were separated through 7.5–15% sodium dodecyl sulfate-polyacrylamide gel electrophoresis. The proteins separated on the gels were transferred onto polyvinylidene difluoride (PVDF) membranes (EMD Millipore, Hayward, CA, USA). The blots were blocked with 5% skim milk solution at room temperature for 1 h and then immunolabeled with primary antibodies (dilution 1:2000–1:10,000) overnight at 4 °C as previously described [53]. The membranes were washed using Tris-buffered saline with 1 × Tween-20 and then incubated with horseradish peroxidase-conjugated secondary antibodies (dilution 1:3000) at room temperature for 1 h. The immunolabeled protein bands were detected using an enhanced chemiluminescence reagent (Bio-Rad Laboratories, Hercules, CA, USA) according to the manufacturer’s instructions. Band density was analyzed using ImageJ 1.5 software (NIH, Bethesda, MD, USA), and the expression levels were determined as the normalized ratio of each target protein to β-actin.

### 4.8. Statistical Analysis

The results are expressed as the mean ± standard deviation from at least three independent experiments. Statistical analysis was performed by analysis of variance with Tukey’s post hoc test using SPSS 9.0 software (SPSS Inc., Chicago, IL, USA). Statistical significance was considered at *p* < 0.05.

## 5. Conclusions

In this study, we investigated the anticancer effects and underlying the molecular mechanisms of the natural CypA inhibitors, C9 and CsA, on NSCLC CSCs. C9 and CsA significantly inhibited the proliferation and tumorsphere formation of NSCLC CSCs and NSCLC CSC-derived tumor growth in vivo by activating the intrinsic apoptotic pathway. Notably, both compounds suppressed the expression of major CSC markers, such as integrin α6, CD133, CD44, ALDH1A1, Nanog, Oct4, and Sox2, through the dual downregulation of CypA/CD147 and EGFR in NSCLC CSCs. In addition, combined treatment with afatinib and C9 or CsA was more potent than single-compound treatment at inhibiting the growth of EGFR-mutant NSCLC CSCs. These findings suggest that the natural CypA inhibitors C9 and CsA may be attractive anticancer agents that effectively suppress the growth of EGFR-mutant NSCLC CSCs by interfering with the crosstalk between the CypA/CD147 and EGFR pathways.

## Figures and Tables

**Table 1:** IC_50_ values of C9 and CsA on the proliferation of NSCLC CSCs.

Compounds	IC_50_ Values (µM)			
	A549 CSCs	NCI-H1299 CSCs	NCI-H1650 CSCs	HCC827 CSCs
C9	49.03 ± 0.015	57.56 ± 0.036	29.18 ± 0.027	25.57 ± 0.014
CsA	9.34 ± 0.051	9.72 ± 0.059	2.47 ± 0.035	8.90 ± 0.009

## Data Availability

The data that support the findings of this study are available from the corresponding author upon reasonable request.

## Supplementary Materials

The supporting information can be downloaded at: https://www.mdpi.com/article/10.3390/ijms24119437/s1**.**
