# Supplementary material for: Natural Cyclophilin A Inhibitors Suppress the Growth of Cancer Stem Cells in Non-Small Cell Lung Cancer by Disrupting Crosstalk between CypA/CD147 and EGFR
Source: Int J Mol Sci. 2023 May 29;24(11):9437. doi: 10.3390/ijms24119437 (PMC10253869; doi:10.3390/ijms24119437)
Supplement: Supplementary file 1 [file ijms-24-09437-s001.zip › ijms-2358422-supplementary.pdf]

Article

Natural Cyclophilin A Inhibitors Suppress the Growth of Cancer Stem Cells in Non-Small Cell Lung Cancer by Disrupting Crosstalk between CypA/CD147 and EGFR

Jang Mi Han [1], Sung Min Kim [1], Hong Lae Kim [2], Hee Jeong Cho [1] and Hye Jin Jung [1,2,3,*]

[1] Department of Life Science and Biochemical Engineering, Graduate School, Sun Moon University, Asan 31460, Republic of Korea; gkswkdal200@naver.com (J.M.H.); tjdals8855@gmail.com (S.M.K.); tjdrjdh@naver.com (H.J.C.)
[2] Department of Pharmaceutical Engineering and Biotechnology, Sun Moon University, Asan 31460, Republic of Korea; llee5405@gmail.com
[3] Genome-Based BioIT Convergence Institute, Sun Moon University, Asan 31460, Republic of Korea
* Correspondence: poka96@sunmoon.ac.kr; Tel.: +82-41-530-2354; Fax: +82-41-530-2939

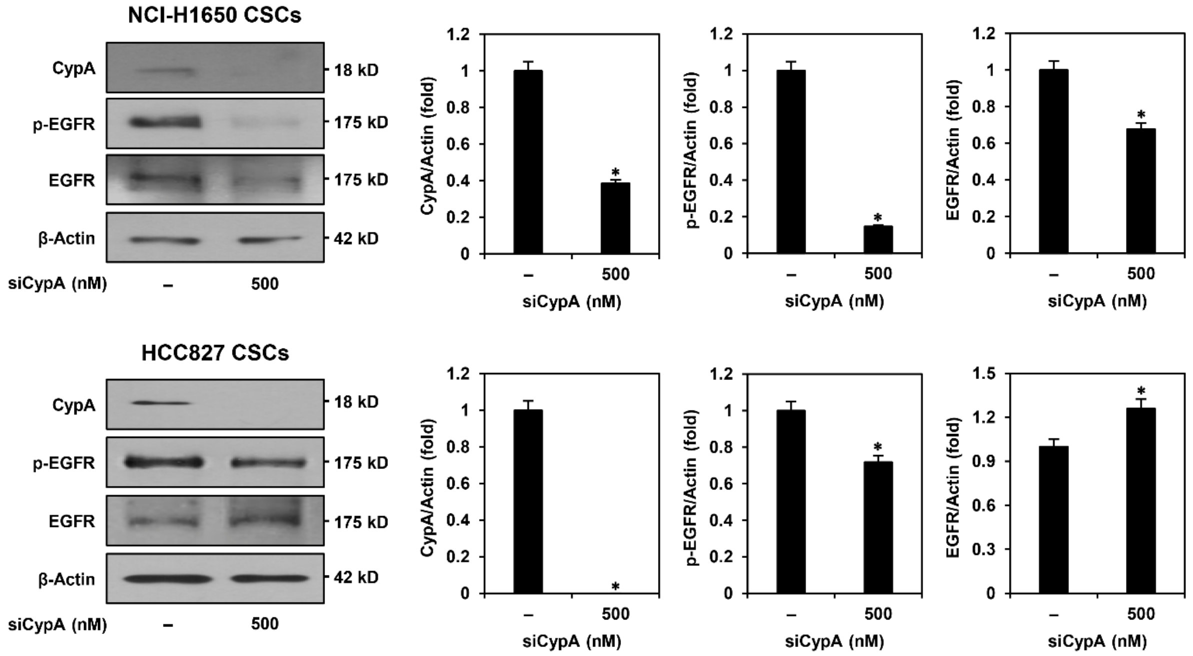

Figure S1. Effect of CypA knockdown on the phosphorylation of EGFR in NCI-H1650- and HCC827-derived NSCLC CSCs. Protein levels were detected by western blot analysis using specific antibodies and were further quantified by densitometry. β-Actin levels were used as an internal control. * $p < 0.05$ vs. the control group.

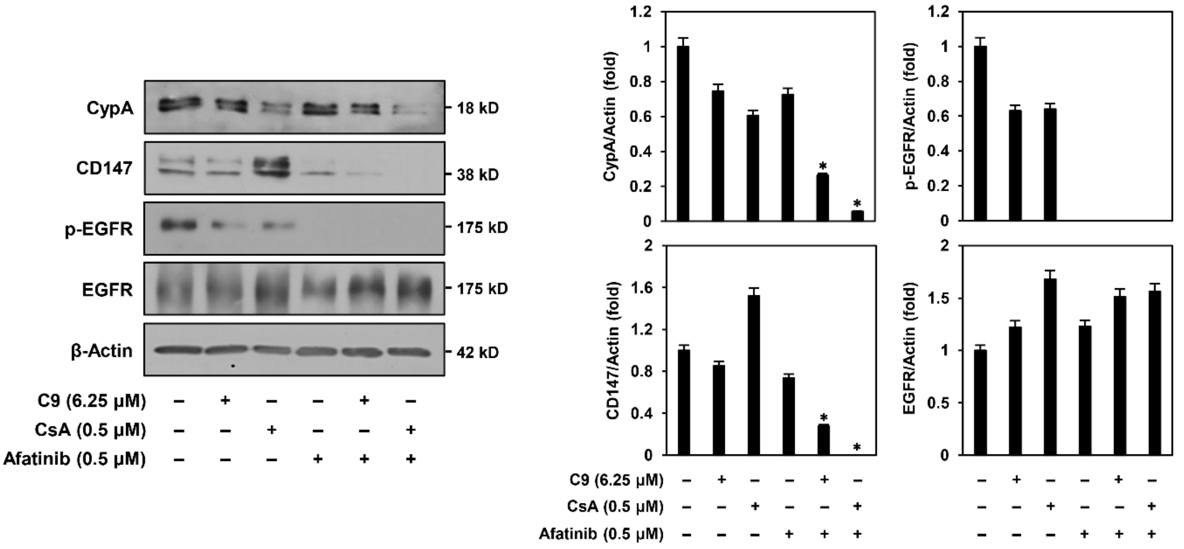

Figure S2. Effect of combined treatment with CypA inhibitors and afatinib on the expression levels of CypA, CD147, and p-EGFR in NCI-H1650-derived NSCLC CSCs. Protein levels were detected by western blot analysis using specific antibodies and were further quantified by densitometry. β-Actin levels were used as an internal control. * $p < 0.05$ vs. the compound alone.
